# Supplementary material for: Lung Inflammation Induced by Inactivated SARS-CoV-2 in C57BL/6 Female Mice Is Controlled by Intranasal Instillation of Vitamin D
Source: Cells. 2023 Apr 6;12(7):1092. doi: 10.3390/cells12071092 (PMC10093523; doi:10.3390/cells12071092)
Supplement: Supplementary file 1 [file cells-12-01092-s001.zip › cells-2247374-supplementary Table S1.pdf]

Supplementary Table S1: Clones of antibodies used in flow cytometry for immunophenotyping of lung cells.

Antibody	Manufacturer	Clone	Antibody	Manufacturer	Clone
CD45	Biolegend	103151	F4/80	eBiosciences	BM8
LD	Invitrogen	N/A	IFN-γ	Biolegend	XMG1.2
CD103	eBiosciences	46-1031-82	IL-17	eBiosciences	Ebio 17B7
CD19	BD Biosciences	HIB19	TNF-α	eBiosciences	MP6-XT22
CD11b	Biolegend	101257	IL-6	Biolegend	MP5-20F3
Ly6G	Biolegend	127626	Thy 1.2	BD Biosciences	105331
CD11c	Biolegend	117336	TCR-β	BD Biosciences	47-5961-82
MHC II	Biolegend	M5/114.15.2	FC Block (anti CD16/32)	Bioxcell	2.4G2
Ly6C	Biolegend	HK1.4	SinglecF	BD Biosciences	562757
CD64	Biolegend	X54-5/7-1			
