# Supplementary material for: Lung Inflammation Induced by Inactivated SARS-CoV-2 in C57BL/6 Female Mice Is Controlled by Intranasal Instillation of Vitamin D
Source: Cells. 2023 Apr 6;12(7):1092. doi: 10.3390/cells12071092 (PMC10093523; doi:10.3390/cells12071092)
Supplement: Supplementary file 1 [file cells-12-01092-s001.zip › Supplementary Figure S1.pdf]

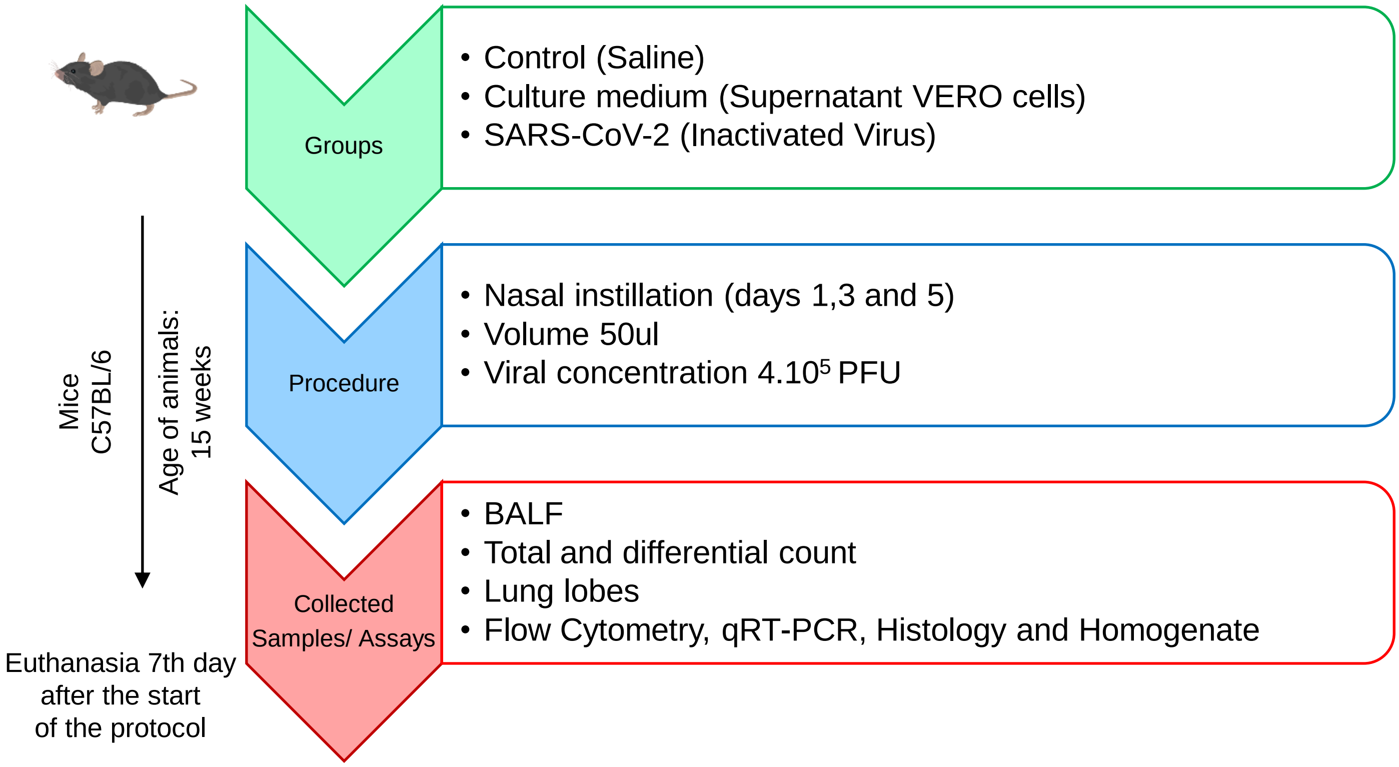
Mice
C57BL/6
Age of animals:
15 weeks
Euthanasia 7th day
after the start
of the protocol
Groups
• Control (Saline)
• Culture medium (Supernatant VERO cells)
• SARS-CoV-2 (Inactivated Virus)
Procedure
• Nasal instillation (days 1,3 and 5)
• Volume 50ul
• Viral concentration 4.10^5 PFU
Collected
Samples/ Assays
• BALF
• Total and differential count
• Lung lobes
• Flow Cytometry, qRT-PCR, Histology and Homogenate

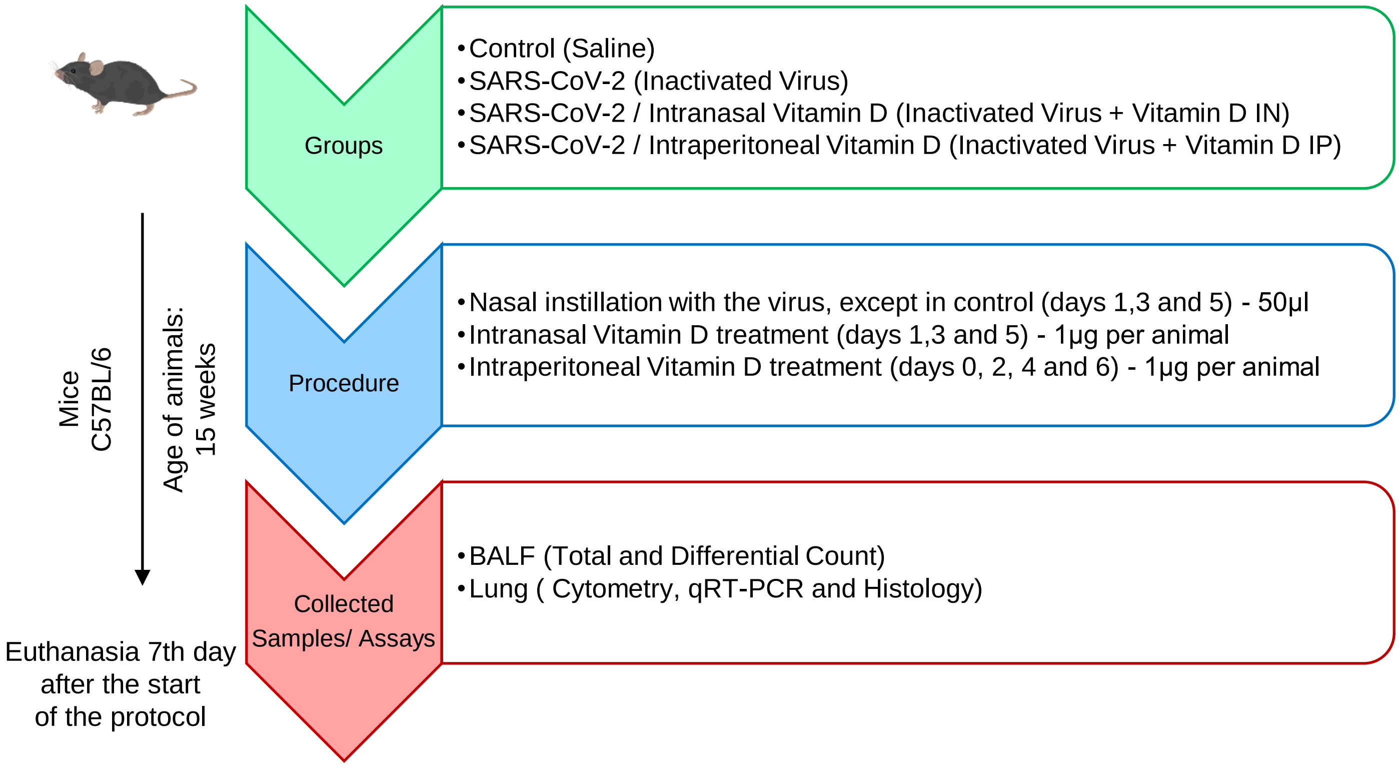
Mice
C57BL/6
Age of animals:
15 weeks
Euthanasia 7th day
after the start
of the protocol
Groups
• Control (Saline)
• SARS-CoV-2 (Inactivated Virus)
• SARS-CoV-2 / Intranasal Vitamin D (Inactivated Virus + Vitamin D IN)
• SARS-CoV-2 / Intraperitoneal Vitamin D (Inactivated Virus + Vitamin D IP)
Procedure
• Nasal instillation with the virus, except in control (days 1,3 and 5) - 50µl
• Intranasal Vitamin D treatment (days 1,3 and 5) - 1µg per animal
• Intraperitoneal Vitamin D treatment (days 0, 2, 4 and 6) - 1µg per animal
Collected
Samples/ Assays
• BALF (Total and Differential Count)
• Lung (Cytometry, qRT-PCR and Histology)
