# Supplementary figures and images for: Lung Inflammation Induced by Inactivated SARS-CoV-2 in C57BL/6 Female Mice Is Controlled by Intranasal Instillation of Vitamin D
Source: Cells. 2023 Apr 6;12(7):1092. doi: 10.3390/cells12071092 (PMC10093523; doi:10.3390/cells12071092)

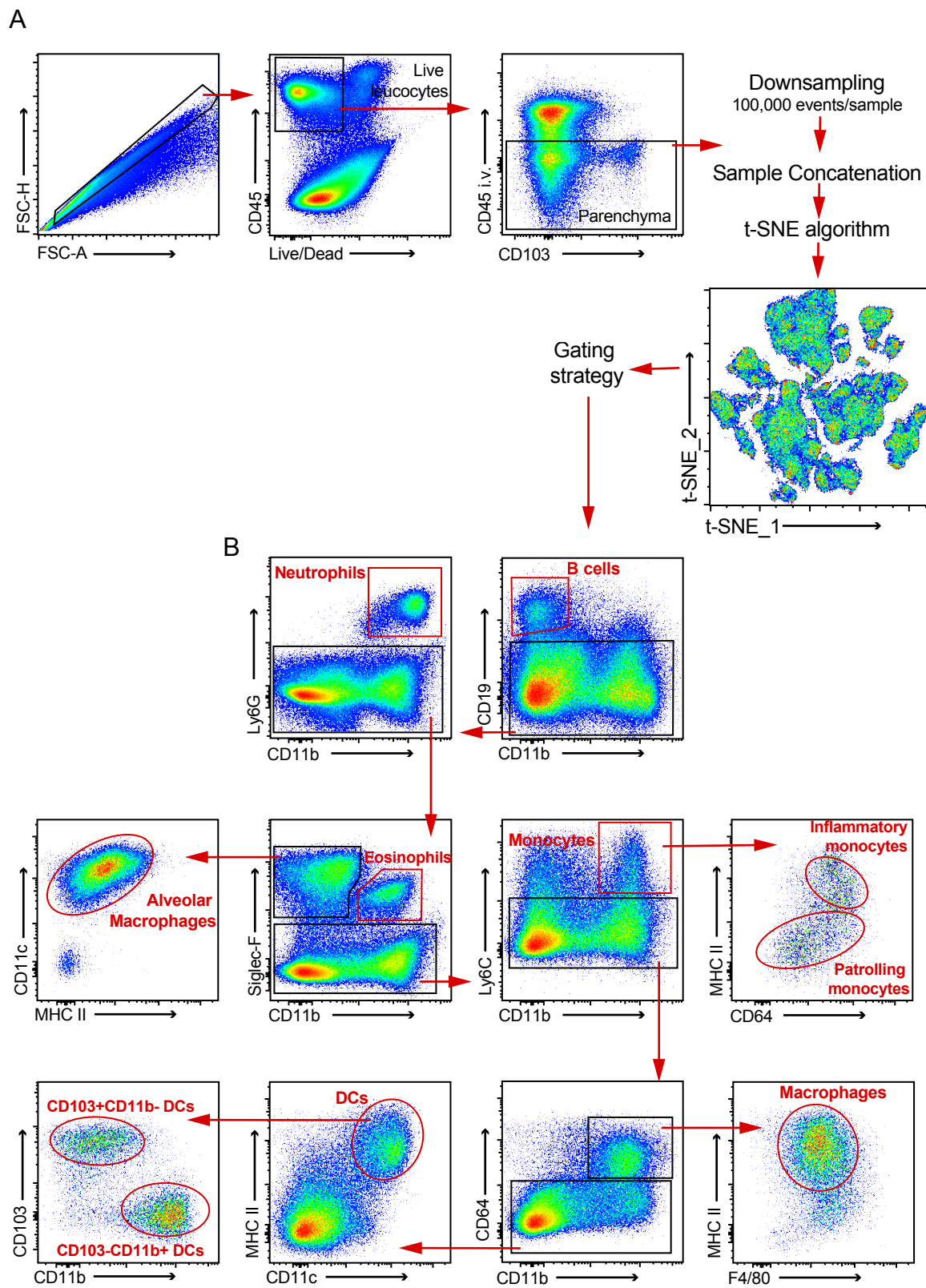

Supplement: Supplementary file 1 [file cells-12-01092-s001.zip › Supplementary Figure S2.pdf]

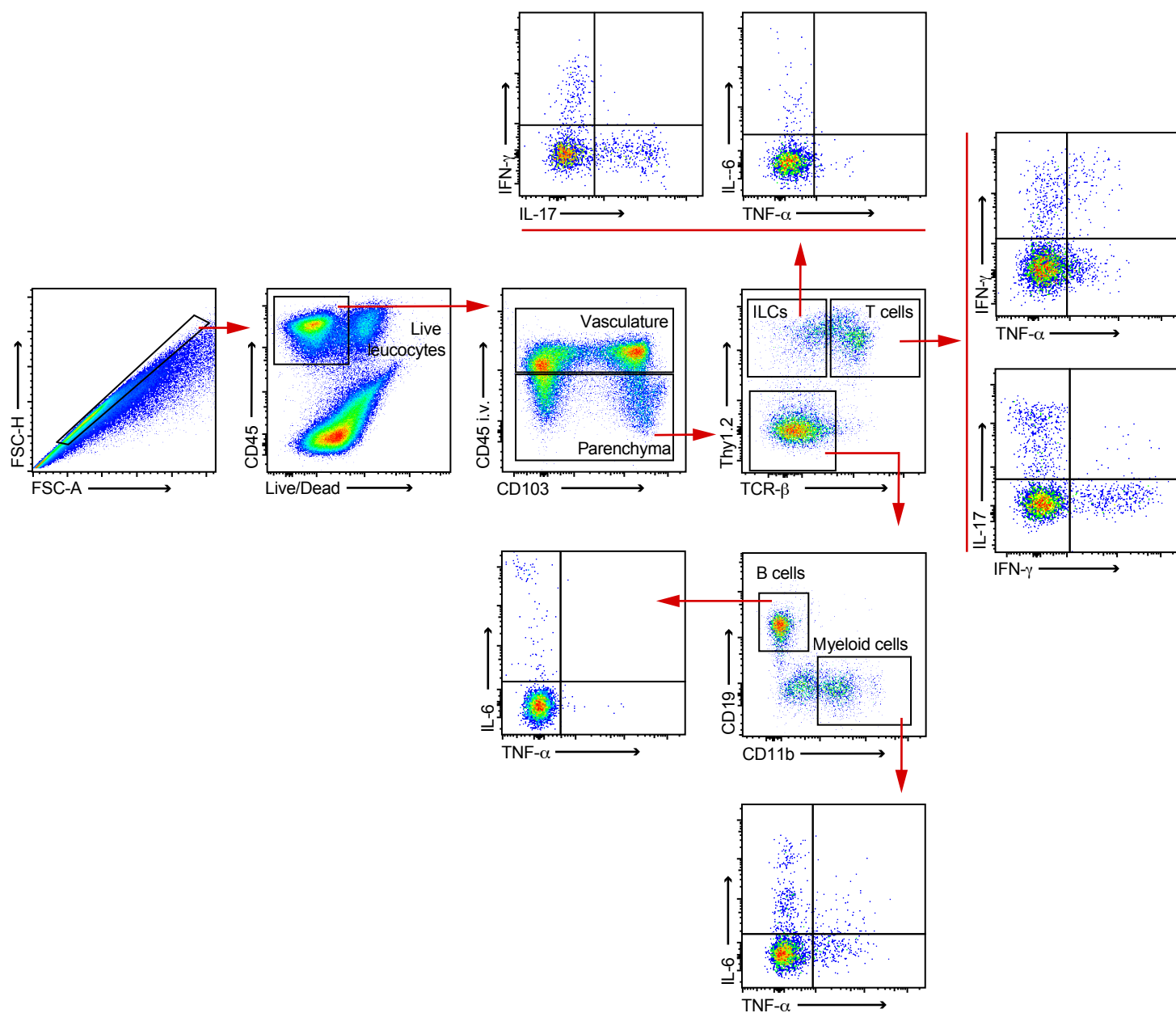

Supplementary Figure 3

Supplement: Supplementary file 1 [file cells-12-01092-s001.zip › Supplementary Figure S3.pdf]

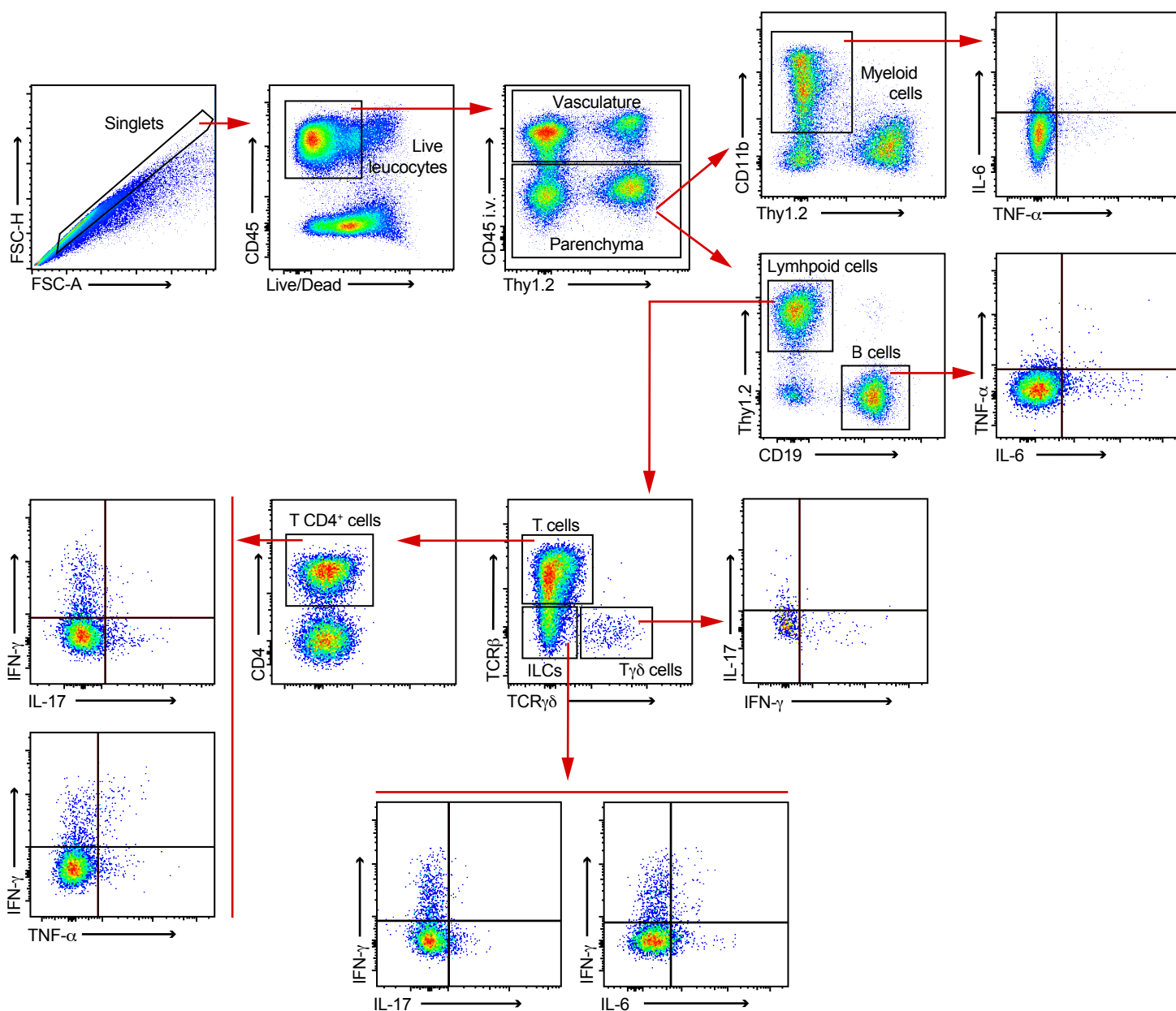

Supplementary Figure 4

Supplement: Supplementary file 1 [file cells-12-01092-s001.zip › Supplementary Figure S4.pdf]
